# Obstacles au don bénévole de sang dans la population de Kisangani en République Démocratique du Congo

**DOI:** 10.11604/pamj.2014.17.306.2663

**Published:** 2014-04-22

**Authors:** Salomon Batina Agasa, Joris Losimba Likwela

**Affiliations:** 1 Faculté de Médecine, Université de Kisangani, B.P. 2012, Kisangani, République Démocratique du Congo

**Keywords:** Transfusion, don de sang, Connaissance, attitudes, pratiques, République Démocratique du Congo, Transfusion, Blood donation, knowledge, attitudes, practices, Democratic Republic of Congo

## Abstract

**Introduction:**

Plusieurs facteurs font obstacles au don de sang dans les pays en développement. La présente étude avait pour objectifs d'analyser les connaissances, attitudes et pratiques de la population de Kisangani relatives au don de sang et d'identifier les obstacles au don de sang afin de guider la planification des activités de promotion du don de sang.

**Méthodes:**

L’ enquête a été réalisée du 4 février au 27 août 2008 sur un échantillon de 1067 sujets par entretien.

**Résultats:**

Sur 1067 sujets interviewés, 1002 (93.9%) sujets ont accepté de répondre. La plupart de nos enquêtés (81.5%, n=1002) avait connaissance de la pratique du don bénévole de sang à Kisangani. Quoique la majorité des sujets approuvent la pratique du don de sang, 57.9% (n=416) parmi eux n'ont jamais fait un don de sang. La raison principale avancé par ceux qui n'ont jamais fait un don de sang était qu'ils n'avaient pas été sollicités tandis que 35% (n=144), quoique sollicités, avaient refusés de faire un don de sang pour diverses raisons.

**Conclusion:**

Cette enquête met en lumière le fait qu'il y a maintenant dans la population de Kisangani une attitude favorable au don de sang. Mais plusieurs obstacles empêchent encore le passage à l'acte. Des activités de promotion qui s'appuient sur la communication interpersonnelle devraient être mises sur pieds afin de passer des messages personnalisés aux donneurs potentiels de sang.

## Introduction

Assurer l'autosuffisance en sang de qualité sur toute l’étendue de son territoire est le but de la politique nationale de la transfusion sanguine dont s'est doté la République Démocratique du Congo (RDC) depuis novembre 1999. Pour atteindre cet objectif, développer un réseau des structures décentralisées avec entre autres des Centres Provinciaux de Transfusion Sanguine (CPTS), promouvoir le don volontaire et non rémunéré de sang font partie des stratégies utilisées.

Cependant, l'offre est encore loin de couvrir la demande en sang. A Kisangani, ville du nord-est avec une population estimée à 900 000 habitants, le CPTS couvre environ 30% des besoins. Cette situation est caractéristique de la région africaine de l'OMS où une évaluation de satisfaction des besoins en sang réalisée en 2006 avait relevé seulement 41.5% de besoins couverts [1]. Plusieurs facteurs relatifs aux connaissances, attitudes et pratiques ont été identifiés comme obstacles au don de sang dans les pays en développement [2–6]. L'exploration de ces facteurs est déterminante pour orienter les stratégies de communication et de promotion de la pratique du don de sang. Une revue de la littérature réalisée entre avril et mai 2011 n'a relevé aucune étude sur les connaissances, attitudes et pratiques (CAP) sur le don de sang en RDC [2].

La présente étude avait pour objectifs d'analyser les connaissances, attitudes et pratiques de la population de Kisangani relatives au don de sang et d'identifier les obstacles au don de sang afin de guider la planification des activités de promotion du don de sang.

## Méthodes

L'enquête, réalisée du 4 février au 27 août 2008, a porté sur les sujets âgés de 18 à 65 ans sélectionnés par échantillonnage à 3 degrés: échantillonnage aléatoire simple de 4 quartiers dans chacune des six communes de la ville (Kabondo, Kisangani, Lubunga, Makiso, Mangobo et Tshopo), échantillonnage aléatoire simple de 7 avenues dans chaque quartier sélectionné, puis échantillonnage systématique des ménages proportionnel à la taille des avenues. Dans chaque ménage le chef de ménage ou un autre membre de la famille présent (épouse, enfant âgé de 18 ans à 65 ans) était interviewé.

La taille de l’échantillon était calculée selon la formule: *n=Z*^*2*^_*α/2*_(p^*^q)/d^*^2^*^

Pour une taille maximale d’échantillon, en l'absence de données d’études CAP en RDC, afin d'estimer la proportion de sujets favorables ou non au don de sang, nous avons retenu p=50%. En considérant un effet de grappe de 2 et un risque d'erreur de 3%, la taille de l’échantillon était de 1067 sujets.

Un questionnaire validé par des experts (un spécialiste en médecine transfusionnelle, des prestataires du CPTS et le responsable de l'association des donneurs bénévoles de Kisangani) a été utilisé après traduction en langue locale, pour les sujets ne pouvant répondre en français, et pré-test dans un quartier qui a été exclu du processus de sélection de l’échantillon. Le questionnaire a servi de guide d'entretien.

Les questions portaient sur le fait d'avoir déjà entendu parlé de la pratique du don de sang à Kisangani, la source d'information, l'approbation ou non de la pratique du don de sang, le fait d'avoir déjà donné son sang, le fait d'avoir déjà été sollicité pour le don de sang et les raisons d'une opposition au don de sang. Les variables sociodémographiques considérées étaient l’âge, le sexe, le niveau d’étude, la confession religieuse et le lieu de résidence.

## Résultats

Le Table 1 présente les caractéristiques socio-économiques des enquêtés. Sur 1067 questionnaires soumis, 1002 (93.9%) sujets ont répondu, parmi lesquels 714 (71.3%) étaient du sexe masculin, près du tiers d'enquêtés étaient sans emploi rémunéré (30.1%) et la majorité (84.5%) du niveau secondaire ou supérieur. L’âge médian était de 28.8 ans. Les répondants étaient principalement de religion protestante (34.2%), des églises dites de réveil (28%) ou de religion catholiques (23.4%) et près de 3% étaient des témoins de Jéhovah.

**Table 1:** Répartition des enquêtés selon les caractéristiques socio-démographiques

Caractéristiques sociodémographiques	n (=1002)	%*
**Age en années : Médiane (EIQ)**		28.5 (21.5 -35.5)
**Sexe**		
Féminin	288	28.7
Masculin	714	71.3
**Profession**		
Elèves, étudiants	410	40.9
Sans emploi	302	30.1
Salariés	290	29
**Niveau d’étude**		
secondaire	541	54
supérieur et universitaire	306	30.5
primaire	92	9.2
analphabète	63	6.3
**Religion**		
Protestant	343	34.2
Eglise de réveil	281	28
Catholique	234	23.4
Kimbanguiste	71	7.1
Musulmane	44	4.4
Témoins de Jéhovah	29	2.9

* Sauf pour l’âge

**Figure 1:**
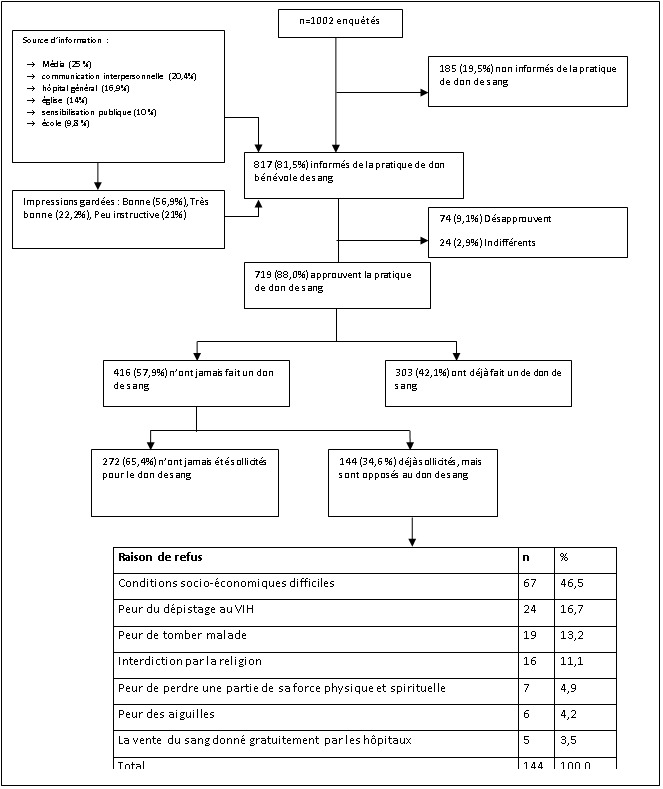
Attitude et pratiques des enquêtés face au don de sang

La Figure 1 présente l'attitude des enquêtés en rapport avec le don de sang. La plupart de nos enquêtés (81.5%, n=1002) avait connaissance de la pratique du don bénévole de sang à Kisangani. Quoique la majorité des sujets approuvent la pratique du don de sang, 57.9% (n=719) parmi eux n'ont jamais fait un don de sang. La raison principale avancé par ceux qui n'ont jamais fait un don de sang était qu'ils n'avaient pas été sollicités tandis que 35% (n=416), quoique sollicités, avaient refusés de faire un don de sang pour diverses raisons.

## Discussions

La présente étude montre que la pratique du don bénévole de sang est assez largement connue de la population. L'information leur parvient à travers les médias, la communication interpersonnelle, les hôpitaux, les églises, les sensibilisations sur les places publiques et les écoles. Kisangani, capitale de la province Orientale, est le site d'implantation du CPTS et fait l'objet de multiples approches de sensibilisation pour mobiliser des donneurs bénévoles de sang. Dans cette étude, seule une personne sur cinq avait fait l'objet d'une communication interpersonnelle. Dans une enquête française au cours de laquelle il était demandé aux sujets “pourquoi ne donnez-vous pas le sang ?” les deux raisons majeures avancées pour n’‘avoir pas donné le sang étaient “parce que je n'y ai pas pensé” (34%) et “parce que personne ne me l'a demandé” (32%) [6]. Des enquêtes menées au Pakistan, en Arabie Saoudite et à Trinidad et Tobago avaient également relevé comme principale raison de ne pas donner du sang le fait que personne ne l'avait demandé aux enquêtés [2]. Dans notre série, bien que la majorité d'enquêtés approuvait cette pratique (88%), parmi ceux qui n'ont jamais fait un don de sang, la grande majorité (65.4%) disait ne l'avoir jamais fait pour n'avoir pas encore été sollicités.

Les conditions socio-économiques difficiles ont constitué près de la moitié des raisons de refus de donner bénévolement du sang. Cette raison qui semble dépendre en grande partie du pouvoir que l'on attribue au sang sur la santé d'un individu, est diversement motivée: les uns craignent qu'un don éventuel de sang déséquilibre leur état de santé qu'ils estiment fragile par leur condition de vie; les autres pensent qu'en étant dans ces situations, leur don devrait plutôt être rémunéré afin de leur permettre d'accéder à une alimentation capable de « reconstituer » la quantité de sang donné.

Le don rémunéré persiste encore à côté du don familial qui est la principale modalité de don de sang observées en Afrique subsaharienne [7]. À Kisangani, en RDC, il a été rapporté que 50% des donneurs rémunérés étaient séropositifs au VIH, 64.3% porteurs de l'AgHBs et 63,6% de marqueurs de la syphilis [8]. Ces fréquences élevées des marqueurs infectieux, supérieures à celles observées chez les donneurs familiaux (4.6% pour VIH, 4.9% pour AgHBs et 3.6% pour la syphilis) et de loin encore à celles des donneurs bénévoles (2.2% pour VIH, 3% pour AgHBs et 1% pour la syphilis), montrent le risque accru de transmission d'infections par transfusion si l'on devait recourir à cette catégorie de donneurs [8].

Diverses « peurs » ont constitué près du tiers des motifs de refus de donner du sang: peur du dépistage du VIH, peur de tomber malade, peur de perdre la force physique et spirituelle et peur des aiguilles. Plusieurs autres études africaines ont relevé des facteurs similaires comme obstacles au don bénévole de sang [3–6, 9]. Autant de facteurs à prendre en compte dans la communication interpersonnel afin d'amener les personnes, par ailleurs favorables au don, à passer à l'action.

Comme dans d'autres pays d'Afrique, les convictions religieuses étaient une raison de ne pas donner du sang pour 11,1% des sujets d'enquête [2, 3, 5]. Outre les témoins de Jéhovah, les croyances religieuses ont été invoquées aussi par des fidèles de certaines sectes religieuses appelées communément "églises de réveil". Le rôle hostile de ces dernières avait déjà été évoqué au Nigeria [10]. Toutefois bien d'autres croyants ont été favorables au don de sang et à la transfusion sanguine. Les chefs religieux devraient être mis à contribution pour l'intensification de la sensibilisation au don bénévole de sang.

Enfin la vente, par les hôpitaux, du sang donné gratuitement a été évoquée comme cause du refus de don de sang. Agbovi et collaborateurs avaient relevé cette perception dans une enquête à Lomé [3]. Il faut dire que la collecte, la préparation des composants sanguins, la conservation ainsi que la distribution ont un coût que le CPTS amortit via la cession d'une poche de sang à 9000 Francs Congolais, équivalent à 10 dollars US. C'est d'ailleurs grâce à la subvention de l'Etat et des appuis de différents partenaires que la population peut avoir la poche de sang à ce prix.

## Conclusion

Cette enquête met en lumière le fait qu'il y a maintenant dans la population de Kisangani une attitude favorable au don de sang. Mais plusieurs obstacles empêchent encore le passage à l'acte. Des activités de promotion qui s'appuient sur la communication interpersonnelle devraient être mises sur pieds afin de passer des messages personnalisés aux donneurs potentiels de sang.
